# AgCl/Ag/g-C_3_N_4_ Hybrid Composites: Preparation, Visible Light-Driven Photocatalytic Activity and Mechanism

**DOI:** 10.1007/s40820-015-0076-y

**Published:** 2015-12-11

**Authors:** Yongchao Bao, Kezheng Chen

**Affiliations:** 1 Lab of Functional and Biomedical Nanomaterials, Qingdao University of Science and Technology, Qingdao, 266042 People’s Republic of China; 2 College of Environment and Safety Engineering, Qingdao University of Science and Technology, Qingdao, 266042 People’s Republic of China; 3 College of Materials Science and Engineering, Qingdao University of Science and Technology, Qingdao, 266042 People’s Republic of China

**Keywords:** AgCl/Ag/g-C_3_N_4_, Hybrid, Photocatalytic activity

## Abstract

The ternary plasmonic AgCl/Ag/g-C_3_N_4_ photocatalysts were successfully fabricated by a modified deposition–precipitation method, through which Ag/AgCl nanoparticles (5–15 nm in size) were evenly dispersed on the surface of g-C_3_N_4_. The AgCl/Ag/g-C_3_N_4_ composites exhibited higher photocatalytic activity than Ag/AgCl and g-C_3_N_4_. The enhanced photocatalytic performance could be attributed to an efficient separation of electron–hole pairs through a Z-scheme mechanism, in which Ag nanoparticles acted as charge separation centers.

## Introduction

As a new metal-free semiconductor, polymeric graphitic carbon nitride (g-C_3_N_4_) has been developed to cope with environmental pollutants due to its outstanding mechanical, optical, electronic, and catalytic properties as well as its high thermal and chemical stability [1–4]. However, its practical application is quite limited owing to its appreciable drawbacks, including low specific surface area, high photogenerated electron–hole recombination rate, and the limited range of visible light photo-responses [5, 6]. To tackle these issues, many methods have been proposed, such as doping extraneous elements [7–10], designing porous structures [11–15], depositing noble metals [16–19], and coupling with other semiconductors [20–23]. Although some progresses have been achieved, the light harvesting ability and quantum efficiency of these modified g-C_3_N_4_ systems are still poor.

Noble metal nanoparticles have attracted considerable attention due to their application as active components for the preparation of various efficient visible light photocatalysts. Logar et al. reported Ag/TiO_2_ plasmonic photocatalyst that showed high efficiency for degradation of methyl orange (MO) [24]. Parida et al. developed Au/g-C_3_N_4_ plasmonic photocatalyst with enhanced photocatalytic activity under irradiation of visible light [17]. Besides, Ag/AgCl [25–27], Ag/AgBr [28], and Ag/AgI [29, 30] have also been used as co-catalysts to enhance the photocatalytic activity of semiconductors under visible light irradiation. It is believed that noble metal nanoparticles can act as active sites and play vital roles in effective visible light absorption and subsequent photocatalytic reactions. The possible reason is that noble metal nanoparticles can strongly absorb visible light because of their localized surface plasmon resonance (LSPR), which can be tuned by varying their size, shape, and surroundings [31].

Recently, two types of Ag/AgCl/g-C_3_N_4_ composites were fabricated by Yao et al. [32] and Zhang et al. [33]. The as-prepared products showed efficient photocatalytic degradation activity. However, their precipitation methods not only lack precise control over the morphology and size of final products, but also induce reunion reaction of AgCl [34].

In this work, AgCl/Ag/g-C_3_N_4_ composites were fabricated by a modified deposition–precipitation method to overcome the above shortcomings. The photocatalytic activities of the AgCl/Ag/g-C_3_N_4_ composites were evaluated by the photocatalytic degradation of Rh B and MO aqueous solution under irradiation of visible light. A plasmonic Z-scheme photocatalytic mechanism was then proposed to explain the enhancement of the photocatalytic activity of the AgCl/Ag/g-C_3_N_4_ photocatalysts.

## Experimental

All reagents supplied by Sinopharm Chemical Reagent co., Ltd. are of analytical grade and used as received without further purification.

### Preparation of Photocatalysts

#### Preparation of g-C_3_N_4_ Powders

The metal-free g-C_3_N_4_ powders were fabricated by heating melamine in a muffle furnace. Typically, 5 g of melamine was placed in a semi-closed alumina crucible with a cover. The crucible was heated to 550 °C at a heating rate of 10 °C min^−1^ and held for 4 h. After the reaction, the alumina crucible was cooled to room temperature. The products were collected and ground into powders.

#### Preparation of AgCl/Ag/g-C_3_N_4_ Hybrid Composites

In a typical preparation process, 0.4 g of g-C_3_N_4_ powders and 0.64 g of hexadecyl trimethyl ammonium chloride (CTAC) were added into 200 mL of deionized water, and the suspension was stirred for 30 min and sonicated for 30 min. Then, 4.4 mL of 0.1 M AgNO_3_ was quickly added to the above mixture. During this process, the excessive surfactant CTAC not only adsorbed onto the surface of g-C_3_N_4_ to limit the number of nucleation sites for AgCl to grow, resulting in homogenously dispersed AgCl, but also induced Cl^−^ to precipitate Ag^+^ in the suspension. The resulting suspension was stirred for 1 h and then placed under irradiation of 300 W Xe lamp for 30 min. The suspension was filtered, washed using deionized water, and dried at 80 °C for 8 h. And then, the gray powder was calcined at 300 °C for 3 h. Different molar ratios of AgCl/Ag/g-C_3_N_4_ photocatalysts (3, 5, 10, 15, 20, and 40 at%) were fabricated with the similar procedure.

### Characterization of Photocatalysts

X-ray diffraction (XRD) data were collected on a D-MAX 2500/PC diffractometer (Japan). The surface morphologies of the as-prepared samples were characterized with field emission scanning electron microscopy (FESEM, JEOL JSM-6700F) and transmission electron microscopy (TEM, JEOL JEM-2100F). Photoluminescence (PL) spectra were measured at room temperature on F-4600 fluorescence spectrometer (Hitachi, Japan) with an excitation wavelength of 365 nm. X-ray photoelectron spectroscopy (XPS) analysis was performed on an ESCALAB 250 X-ray photoelectron spectrometer. The Fourier transform infrared spectra (FTIR) of the samples were recorded using IRAffinity-1 spectrometer (Shimadzu, Japan). Ultraviolet visible (UV–Vis) diffuse reflectance spectra (DRS) of the samples were obtained on an UV–vis spectrophotometer (Shimadzu UV-2600, Japan) in the range of 200 to 800 nm and BaSO_4_ as a standard reference. The Brunauer–Emmett–Teller (BET) surface area (S_BET_) of the samples were measured by nitrogen adsorption–desorption isotherm measurements on a micromeritics ASAP2020 system. The electron spin resonance (ESR) signals of ·OH and ·O_2_^−^ radicals spin-trapped with the spin-trap reagent DMPO (5, 5-dimethyl-1-pyrroline-N-oxide) in water and methanol were examined on an ESR spectrometer (ER200-SCR, Germany), respectively.

### Adsorption Experiment

Adsorption experiments were carried out in the dark. In a typical adsorption procedure, 100 mg of 5 at% AgCl/Ag/g-C_3_N_4_ powers mixed with 100 mL 10 mg L^−1^ Rh B aqueous solution in a glass conical beaker was shaken at ambient temperature. At given time intervals, about 3 mL solution suspension was sampled and immediately centrifuged. The concentration of Rh B solution was analyzed with a UV–Vis spectrophotometer at the maximal absorption wavelength of Rh B, whose characteristic absorption peak was chosen to be 554 nm.

### Test of Photocatalytic Activity

The photocatalytic activities of samples were evaluated by the degradation of Rh B and MO under 300 W Xe lamp with a 420 nm cutoff filter. In brief, 100 mg of photocatalyst was dispersed in 100 mL of a 10 mg L^−1^ aqueous solution of Rh B or MO in a reactor with a double layer cooled by running water to keep the temperature unchanged. Prior to irradiation, the suspensions were magnetically stirred in the dark for 1 h to ensure the establishment of an adsorption/desorption equilibrium between the photocatalyst and dye molecules. Then, the suspension was illuminated by the Xe lamp combined with magnetic stirring. At given time intervals, about 3 mL solution suspension was sampled and centrifuged. The concentrations of Rh B and MO were measured by UV–Vis spectrophotometer. Additionally, the recycle experiments were performed for five consecutive cycles to test the durability. After each cycle, the catalyst was centrifuged and washed thoroughly with distilled water several times to remove residual dye impurities and then dried at 80 °C for the next test.

## Results and Discussion

### Characterization of Photocatalysts

**Fig. 1:**
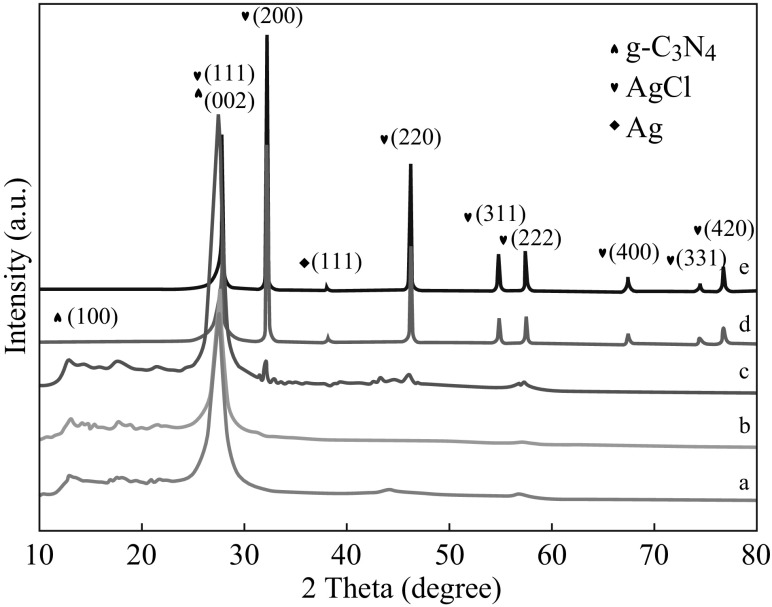
XRD patterns of **a** pure g-C_3_N_4_, **b** 5 at% AgCl/Ag/g-C_3_N_4_ composite, **c** 15 at% AgCl/Ag/g-C_3_N_4_ composite, **d** 20 at% AgCl/Ag/g-C_3_N_4_ composite, and **e** 40 at% AgCl/Ag/g-C_3_N_4_ composite

The crystal structures of the as-prepared samples were analyzed by the XRD pattern. Figure 1 shows the XRD patterns of the pure g-C_3_N_4_ and AgCl/Ag/g-C_3_N_4_ hybrid composites with different Ag/AgCl contents. It is observed that two broad peaks around 27.4° and 13.0° in the XRD patterns of the pure g-C_3_N_4_ are well ascribed to the (002) and (100) diffraction planes, respectively. The former, which corresponds to the interlayer distance of 0.326 nm, is attributed to the long-range interplanar stacking of aromatic units; the latter, with a much weaker intensity, which corresponds to a distance *d* = 0.681 nm, is associated with interlayer stacking [35]. It is obvious that the diffraction peaks at 27.7°, 32.2°, 46.2°,54.8°,57.5°,67.4°, 74.5°, and 76.7° gradually appear and the intensity increases with the increase of Ag/AgCl content, and the peaks are assigned to the (111), (200), (220), (311), (222), (400), (331), and (420) planes of AgCl crystal, respectively. A weak diffraction peak at 38.2° is also seen, which corresponds to the (111) plane of Ag crystal. It is difficult to distinguish the characteristic peak of g-C_3_N_4_ (27.4°) and the AgCl (111) plane (27.8°) because they are very close to each other. However, with the increase in the amount of Ag/AgCl, the relative intensity of (111)/(200) diffraction of AgCl decreases. This result could be attributed to pure AgCl, the relative intensity of (111) diffraction is about one half of that of (200) diffraction. Therefore, in the XRD analysis, no other crystal phases are observed, which indicated that the AgCl/Ag/g-C_3_N_4_ composites were successfully fabricated.

**Fig. 2:**
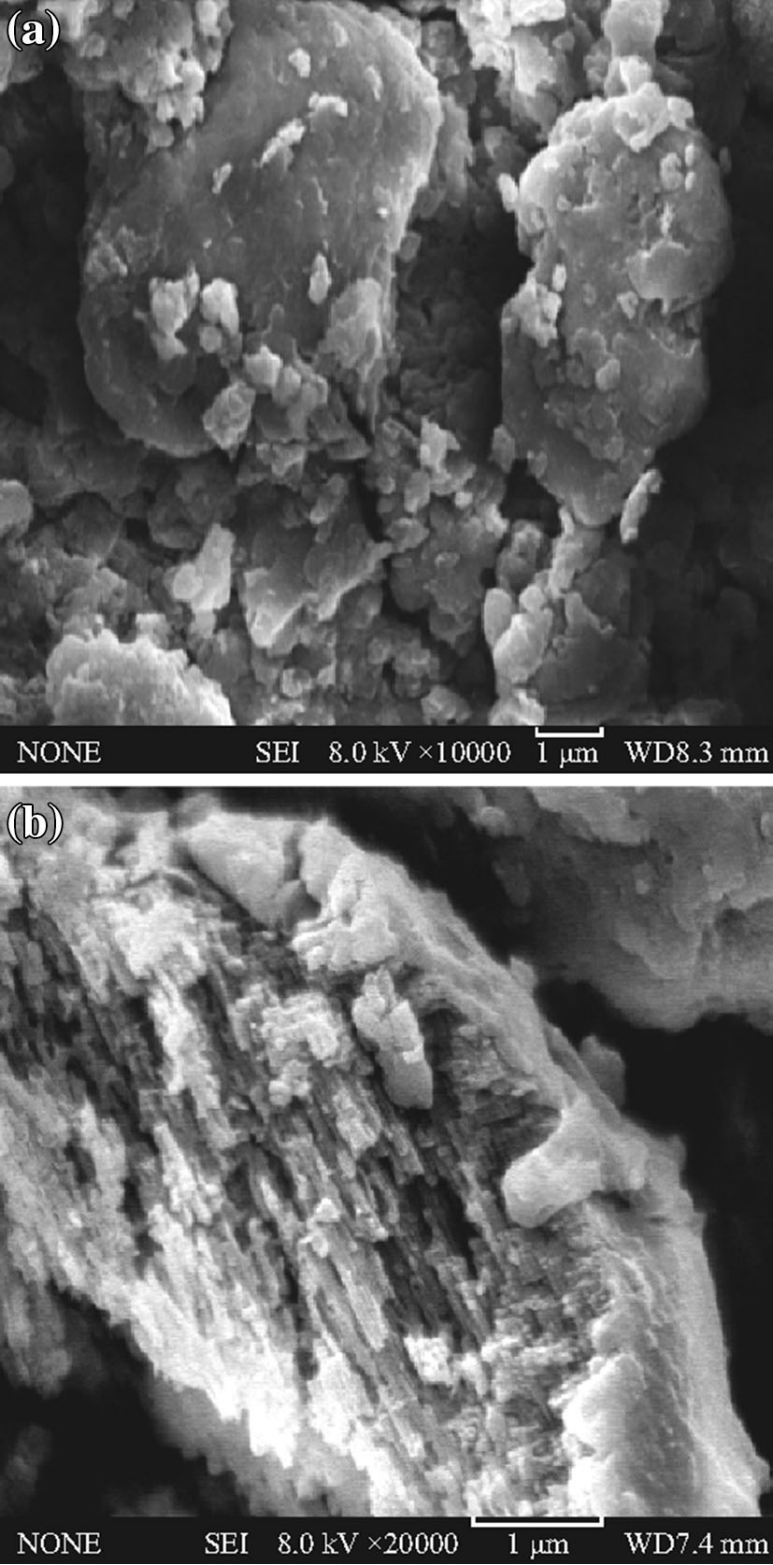
Typical SEM images of **a** pure g-C_3_N_4_ and **b** 5 at% AgCl/Ag/g-C_3_N_4_ composite

**Fig. 3:**
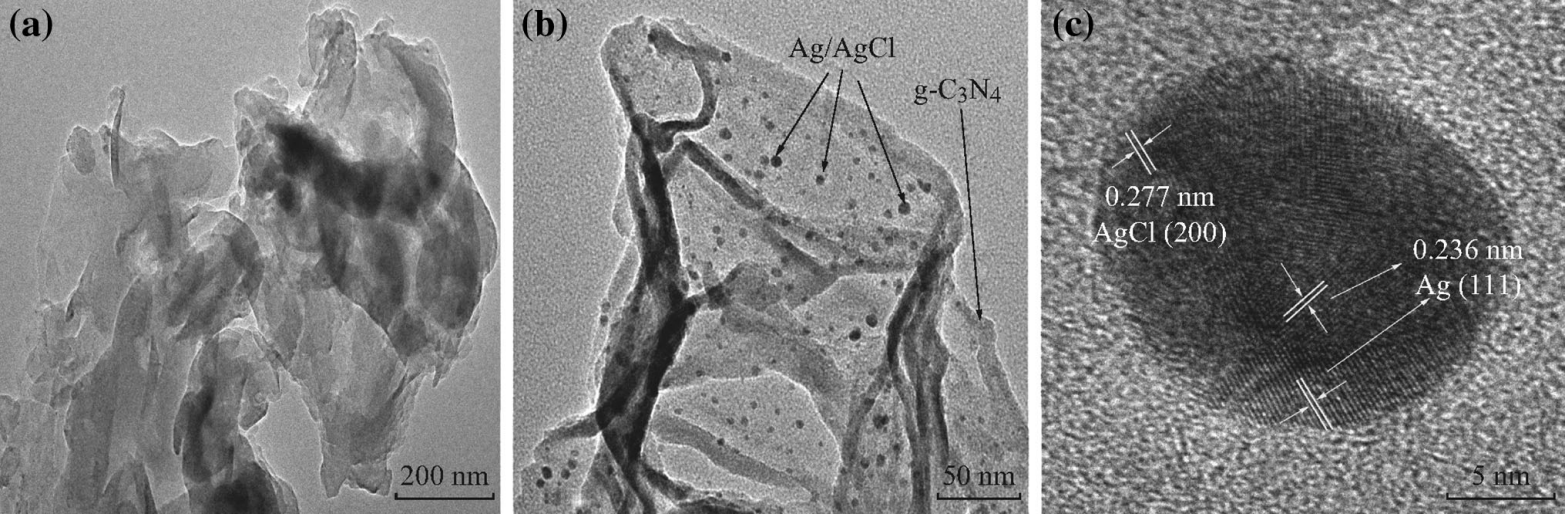
TEM images of **a** pure g-C_3_N_4_ and **b** 5 at% AgCl/Ag/g-C_3_N_4_ composite. **c** HRTEM images of 5 at% AgCl/Ag/g-C_3_N_4_ composite

The morphology and microstructure of the as-prepared samples were investigated by SEM and TEM analysis. Figure 2 shows the SEM images of the pure g-C_3_N_4_ and the 5 at% AgCl/Ag/g-C_3_N_4_ composite. The pure g-C_3_N_4_ sample displays an aggregated morphology with a large size and lamellar structure. The surface of the aggregation is very smooth, showing the layer structure of g-C_3_N_4_. Figure 2b shows Ag/AgCl nanoparticles which might be deposited on the surface of g-C_3_N_4_ and a rough surface was obtained. It is observed from Fig. 3 that the g-C_3_N_4_ displays a lamellar shape and has an amorphous structure, whereas the Ag/AgCl exhibits uniform spherical particles. It is also seen that there are dark spherical particles and gray areas. The dark particles can be assigned to Ag/AgCl, whereas the gray areas can be assigned to g-C_3_N_4_. The Ag/AgCl particles are evenly dispersed on the surface of g-C_3_N_4_, and are approximately 5–15 nm in size. Figure 3c shows that the lattice fringe of 0.236 and 0.277 nm, corresponding to the (111) plane of Ag and (200) plane of AgCl, is clearly observed in the AgCl/Ag/g-C_3_N_4_ composite, which verifies the formation of an AgCl/Ag/g-C_3_N_4_ heterojunction.

**Fig. 4:**
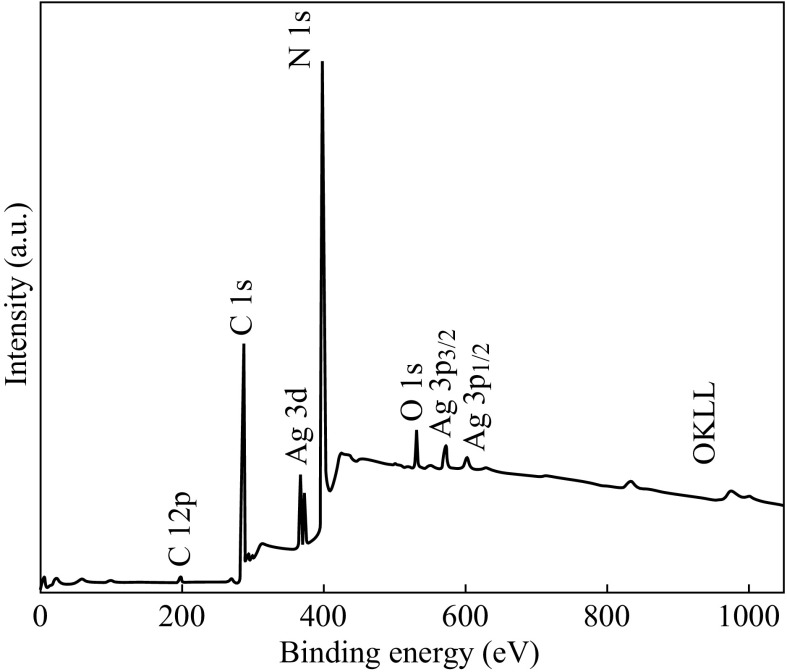
XPS survey spectrum of 5 at% AgCl/Ag/g-C_3_N_4_ composite

**Fig. 5:**
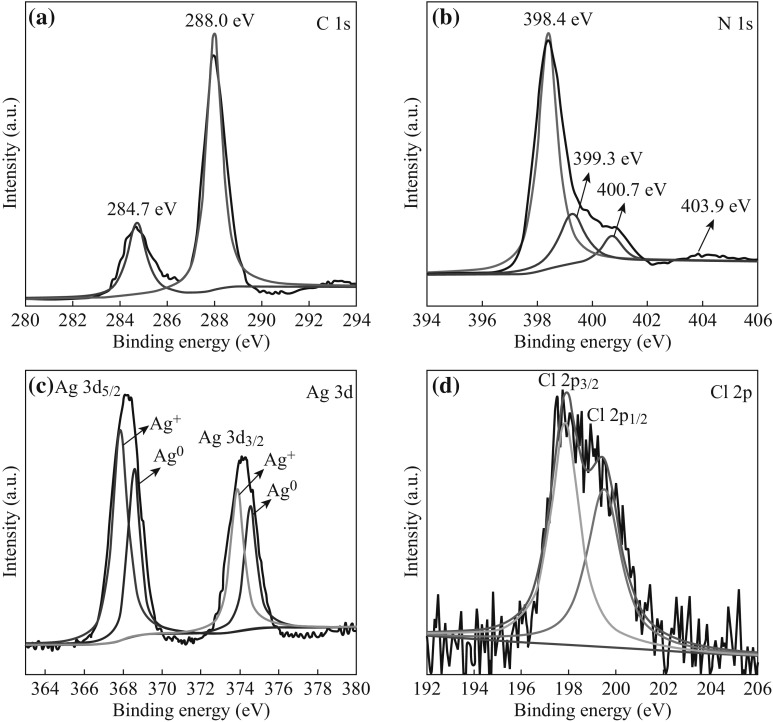
High-resolution XPS spectra of 5 at% AgCl/Ag/g-C_3_N_4_ composite: **a** C1s, **b** N1s, **c** Ag3d, and **d** Cl2p

The chemical composition of the as-prepared 5 at% AgCl/Ag/g-C_3_N_4_ was analyzed by XPS, as shown in Fig. 4. The survey spectrum of 5 at% AgCl/Ag/g-C_3_N_4_ shows peaks of elements of Ag, Cl, C, N, and O. No peaks for other elements is found, indicating that the hybrid composite is primarily composed of Ag, Cl, C, and N elements. To investigate and demonstrate the different chemical states of Ag, C, N, and Cl, the high-resolution XPS peaks of the different elements are provided in Fig. 5. It can be seen from the C1s spectrum that the two C1s peaks are located at 284.7 and 288.0 eV. The former is ascribed to the adventitious contamination and defect-containing *sp* ^2^-hybridized carbon atoms present in graphitic domains, whereas the latter one is assigned to C–N–C coordination [35, 36]. In the N1s spectrum, several binding energies can be separated. The main N1s peak at 398.4 eV corresponds to *sp* ^2^-hybridized aromatic N bonded to C atoms (C=N–C). The peak at 399.3 eV is assigned to the tertiary N bonded to C atoms in the form of N-(C)_3_. The peak at 400.7 eV is from the N–H structure. The weak peak at 403.9 eV is attributed to charging effects [4]. The high-resolution XPS spectra of Ag3d are shown in Fig. 5c. The two peaks at approximately 368.3 and 374.2 eV can be ascribed to the binding energies of Ag3d_5/2_ and Ag3d_3/2_, respectively. These two peaks can be further deconvoluted into two peaks, at about 367.9/368.6 eV and 373.9/374.6 eV, respectively [37]. The peaks at 367.9 and 373.9 eV are attributed to Ag^+^ of AgCl, and those at 368.6 and 374.6 eV are ascribed to the metal Ag^0^. On the basis of XPS peak areas, the mole ratio between Ag^0^ and Ag^+^ was calculated to be 2:3. The Cl2p XPS peaks can also be resolved into two typical peaks, 197.9 and 199.5 eV, which are ascribed to AgCl [38]. All of these results further confirm the coexistence of Ag/AgCl and g-C_3_N_4_ in the AgCl/Ag/g-C_3_N_4_ composite.

**Fig. 6:**
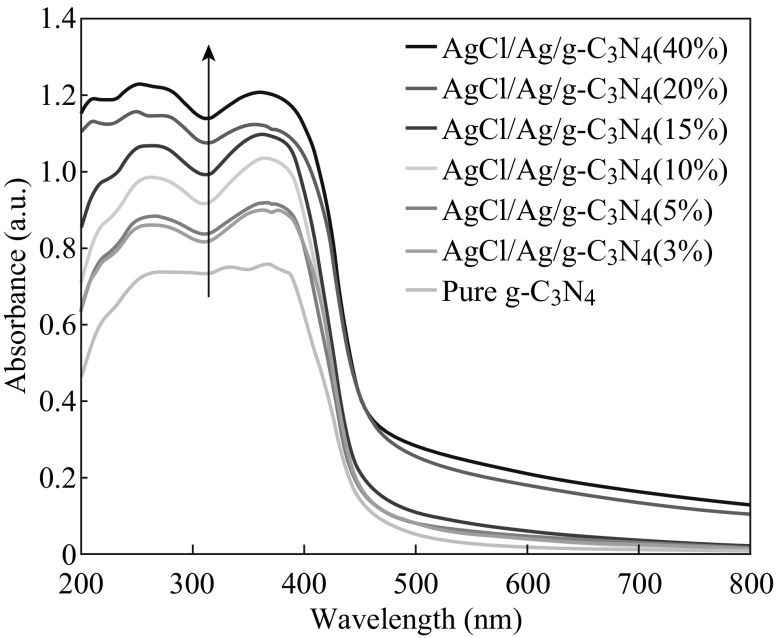
UV-Visible diffuse reflectance spectra of pure g-C_3_N_4_ and AgCl/Ag/g-C_3_N_4_ composites

The UV–Vis diffuse reflectance spectra of the as-prepared samples are shown in Fig. 6. Pure g-C_3_N_4_ has an absorption edge at about 460 nm, which originates from its band gap of 2.7 eV. Compared with the pure g-C_3_N_4_, the series of AgCl/Ag/g-C_3_N_4_ hybrid composites show a slight red shift of the absorption edge, which is attributed to the surface plasmon resonance (SPR) effect of Ag nanocrystal formed in situ on the surfaces of the AgCl nanoparticles. In addition, the absorption intensities of AgCl/Ag/g-C_3_N_4_ composites show a significant enhancement in the visible light regions, which can also be attributed to the SPR effect of Ag nanoparticles.

**Fig. 7:**
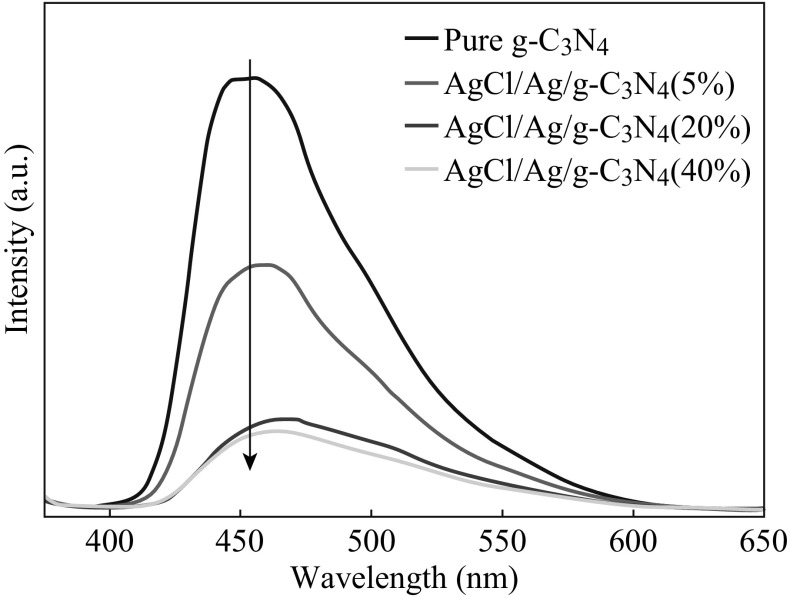
PL spectra of pure g-C_3_N_4_ and AgCl/Ag/g-C_3_N_4_ composites

PL spectra analysis has been widely used to investigate the separation efficiency of photogenerated electrons and holes in semiconductor particles [39]. Figure 7 shows the PL spectra of pure g-C_3_N_4_ and AgCl/Ag/g-C_3_N_4_ composites. It can be found that the strong emission peak of the pure g-C_3_N_4_ centered at 460 nm suggests a high recombination probability of the photogenerated electron–hole pairs, while for AgCl/Ag/g-C_3_N_4_ composites, a significant quenching of PL is observed in comparison with g-C_3_N_4_, which indicates that these composites have lower recombination rates of photogenerated electrons and holes. This demonstrates that after the formation of a heterojunction between g-C_3_N_4_ and Ag/AgCl, the recombination of photogenerated charge carriers is greatly suppressed. Therefore, the photogenerated electron–hole pairs of the AgCl/Ag/g-C_3_N_4_ composites can efficiently transfer at the interface of heterostructure, resulting in the higher photocatalytic activity than pure g-C_3_N_4_ and Ag/AgCl.

**Fig. 8:**
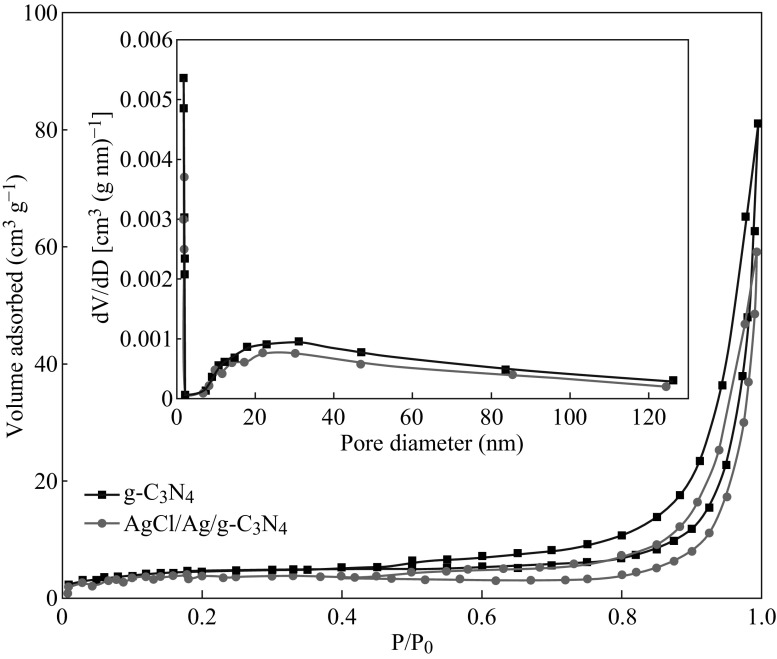
N_2_ adsorption–desorption isotherms of pure g-C_3_N_4_ and 5 at% AgCl/Ag/g-C_3_N_4_ composite. The *inset* shows the corresponding BJH pore size distribution curves of the samples

Full nitrogen adsorption isotherms of pure g-C_3_N_4_ and 5 at% AgCl/Ag/g-C_3_N_4_ composite were measured to gain the information about the specific surface area, as shown in Fig. 8. It is true that the pure g-C_3_N_4_ and the 5 at% AgCl/Ag/g-C_3_N_4_ composites have type IV isotherms and type H3 hysteresis loops, which indicates the mesoporous structure of the samples. The pore size distribution of the samples shows that most of the pores fall into the size range from 3 to 100 nm. The specific surface area of the 5 at% AgCl/Ag/g-C_3_N_4_ composite was calculated to be 11.46 m^3^ g^−1^, which is lower than that of pure g-C_3_N_4_ (14 m^2^ g^−1^). The decrease of the surface area after decoration can be attributed to the disappearance of the small pore, which can be further proved by the pore size distribution shown in the inset of Fig. 8. The results illustrate that the enhanced photocatalytic performance is not a result of the change of the BET surface areas of the samples.

### Adsorption Kinetics

**Fig. 9:**
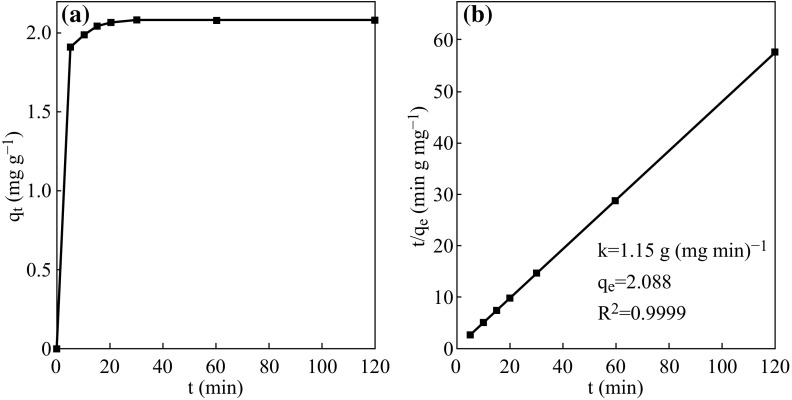
**a** The adsorption-kinetics curve and **b** Pseudo-second-order kinetics of Rh B on 5 at% AgCl/Ag/g-C_3_N_4_ at 25 °C

The adsorption kinetics of Rh B on 5 at% AgCl/Ag/g-C_3_N_4_ is shown in Fig. 9. The adsorption capacity of Rh B increases firstly and then remains unchanged with time. The adsorption equilibrium is achieved after about 30 min. The adsorption kinetics can be well fitted by a pseudo-second-order model, expressed by Eq. (1)

1 $$\frac{t}{{q_{\text{t}} }} = \frac{1}{{kq_{\text{e}}^{2} }} + \frac{t}{{q_{\text{e}} }}$$

where *k* is the rate constant [g (mg min)^−1^], *q*_e_ is equilibrium adsorption capacity (mg g^−1^), and *q*_t_ is the amount of Rh B (mg g^−1^) adsorbed at time *t*. By plotting *t/q*_t_ versus *t*, one can determine the values of *q*_e_ and *k* from the slope and intercept of the fitted line, respectively. The correlation coefficient (*R* ^2^) value of the fitted plot is near unity (0.9999), indicating the applicability of the pseudo-second-order model for the above adsorption kinetics.

### Photocatalytic Activities of the AgCl/Ag/g-C_3_N_4_ Hybrid Composites

**Fig. 10:**
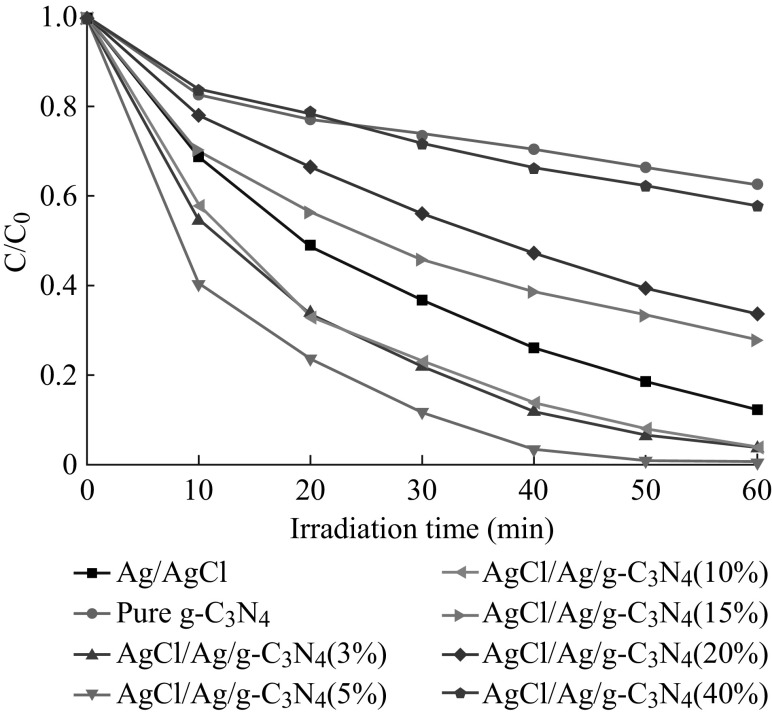
Photocatalytic activities of pure g-C_3_N_4_, Ag/AgCl, and AgCl/Ag/g-C_3_N_4_ composites on the degradation of Rh B under visible light irradiation

The photocatalytic activities of the as-prepared AgCl/Ag/g-C_3_N_4_ composites were evaluated by monitoring the degradation of Rh B in an aqueous solution under visible light irradiation. For comparison, the performances of pure g-C_3_N_4_ and Ag/AgCl photocatalyst were also investigated under same condition. Figure 10 shows the photocatalytic activities of pure g-C_3_N_4_, Ag/AgCl, and AgCl/Ag/g-C_3_N_4_ composites. It can be clearly observed that the photocatalytic activities of AgCl/Ag/g-C_3_N_4_ composites increase firstly and then decrease with the increase of Ag/AgCl content, and the 5 at% AgCl/Ag/g-C_3_N_4_ composite exhibits the highest photocatalytic activity. The decreased photocatalytic activity may be attributed to the fact that the higher content of Ag/AgCl may easily result in the agglomeration of Ag/AgCl particles causing a low dispersibility on the surface of g-C_3_N_4_. This influences the transfer of photogenerated charge carriers. The degradation efficiency of 5 at% AgCl/Ag/g-C_3_N_4_ photocatalyst exceeds 1.14 times more than that of Ag/AgCl and 2.7 times larger than that of g-C_3_N_4_.

**Fig. 11:**
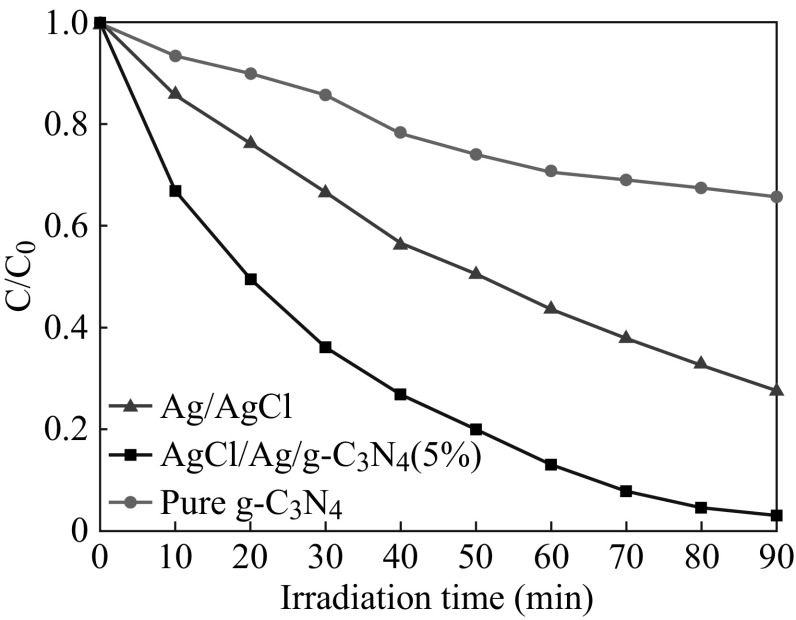
Photocatalytic activities of pure g-C_3_N_4_, Ag/AgCl, and 5 at% AgCl/Ag/g-C_3_N_4_ composite on the degradation of MO under visible light irradiation

The photocatalytic activities of pure g-C_3_N_4_, Ag/AgCl, and 5 at% AgCl/Ag/g-C_3_N_4_ hybrid composite for the degradation of MO were also investigated, as shown in Fig. 11. These results indicate that the photocatalytic activity of 5 at% AgCl/Ag/g-C_3_N_4_ hybrid composite toward MO is also much higher than that of either pure g-C_3_N_4_ or Ag/AgCl. It suggests that a suitable molar ratio between g-C_3_N_4_ and Ag/AgCl is significant for effectively enhancing the photocatalytic activity.

**Fig. 12:**
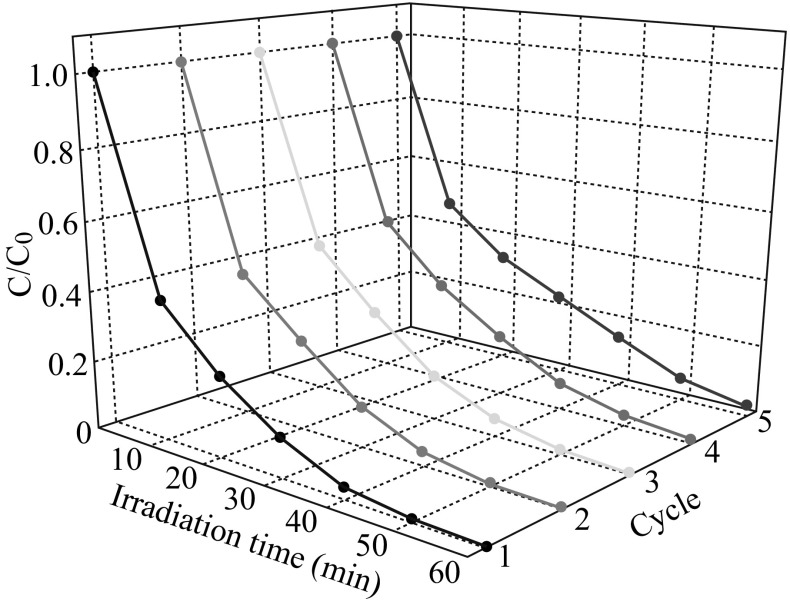
Cycling degradation efficiency of Rh B solution in the presence of 5 at% AgCl/Ag/g-C_3_N_4_ composites under visible light irradiation

Because renewable catalytic was another important factor for a photocatalyst, the stability of the 5 at% AgCl/Ag/g-C_3_N_4_ composite was investigated by a recycling test, as shown in Fig. 12. After five cycles, there was no significant loss of activity, indicating that the photocatalyst was stable during the photocatalytic test.

**Fig. 13:**
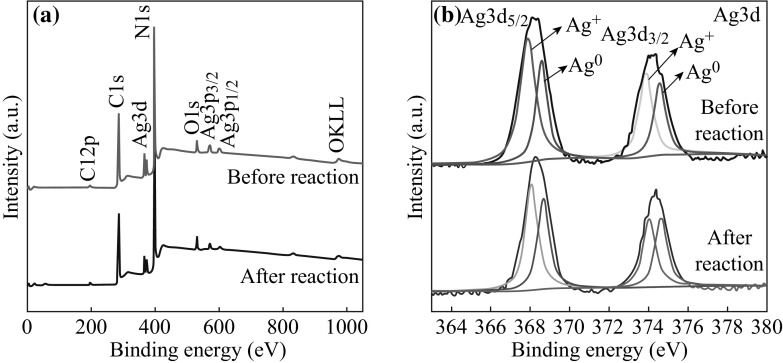
**a** XPS survey spectra of 5 at% AgCl/Ag/g-C_3_N_4_ before and after 5 recycling runs. **b** Ag3d XPS spectra of 5 at% AgCl/Ag/g-C_3_N_4_ before and after 5 recycling runs

The excellent stability was further confirmed by the XPS of 5 at% AgCl/Ag/g-C_3_N_4_ composite before and after photodegradation. Figure 13 shows that there is no noticeable difference between the sample before and after 5 recycling runs. These results indicated that the chemical states of the AgCl/Ag/g-C_3_N_4_ composite surface remained almost unchanged during the photocatalytic degradation of Rh B or MO.

### Photocatalytic Mechanism

**Fig. 14:**
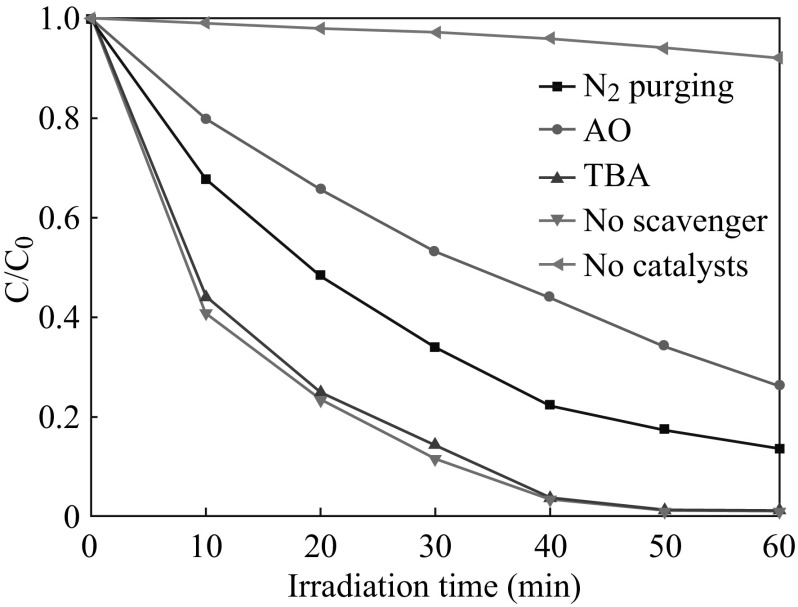
Photocatalytic performances for the degradation of Rh B with 5 at% AgCl/Ag/g-C_3_N_4_ composite with different sacrificial agents under visible light irradiation

To further investigate the photocatalytic mechanism of the AgCl/Ag/g-C_3_N_4_ hybrid composites, a series of radicals trapping experiments were performed by using ammonium oxalate (AO), N_2_ and t-butyl alcohol (TBA) scavengers for holes, ·O_2_^−^ and ·OH radicals, respectively. As is clear from Fig. 14, the addition of TBA did not affect the degradation rate of Rh B over 5 at% AgCl/Ag/g-C_3_N_4_ composite, suggesting that ·OH was not the main reactive species in the photocatalytic process. On the contrary, the photocatalytic degradation of Rh B was obviously suppressed after the addition of AO and N_2_ purging. According to these results, it can be clearly seen that h^+^ and ·O_2_^−^ are main reactive species for 5 at% AgCl/Ag/g-C_3_N_4_ hybrid composite in the photocatalytic degradation process of Rh B under visible light irradiation.

The g-C_3_N_4_ with a band gap of 2.7 eV is a novel metal-free visible light photocatalyst [40]. Under visible light irradiation, the g-C_3_N_4_ absorbs visible light photons to produce photogenerated electrons and holes. The photogenerated electrons react with O_2_ that existed in the photodegradation system, reducing it to superoxide radical anion ·O_2_^−^. The dye molecules are degradated by photogenerated holes and ·O_2_^−^. Ag/AgCl has been demonstrated to be an efficient visible light photocatalyst [31]. As AgCl cannot absorb visible light due to its wide band gap of 3.25 eV, the visible light absorption in Ag/AgCl is attributed to the plasmonic absorption of Ag nanoparticles which can absorb visible light and convert the plasmonic energy into LSPR oscillation. Then, the plasmon-induced electrons from the photoexcited Ag nanoparticles transfer to the CB of AgCl and the electrons on the surface of AgCl are trapped by the adsorbed O_2_ to form ·O_2_^−^ active species, and the plasmon-induced holes stay on the surface of Ag nanoparticles and oxidize the dye molecules [41, 42].

**Fig. 15:**
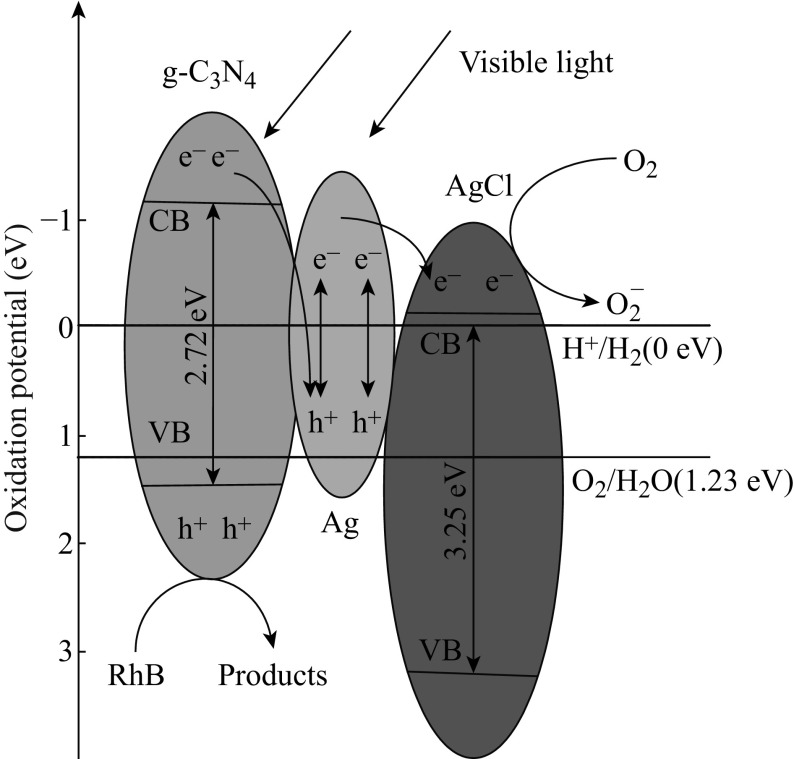
Schematic illustration of the charge separation and transfer in the AgCl/Ag/g-C_3_N_4_ composites under visible light irradiation

When the Ag/AgCl nanoparticles are coupled with g-C_3_N_4_ to form AgCl/Ag/g-C_3_N_4_ composites, a heterojunction structure is formed in the interface between g-C_3_N_4_ sheets and Ag/AgCl nanoparticles. The improved photocatalytic performances are mainly attributed to the separation efficiency of photogenerated electrons and holes in the composites. On the basis of the above results, a plasmonic Z-scheme mechanism of AgCl/Ag/g-C_3_N_4_ composites is proposed and illustrated in Fig. 15. Under visible light irradiation, both Ag and g-C_3_N_4_ absorb visible light photons to produce photogenerated electrons and holes. The plasmon-induced electrons of Ag nanoparticles are transported to the CB of AgCl to reduce oxygen, while the holes remain on the Ag nanoparticles. Meanwhile, the photogenerated electrons of g-C_3_N_4_ transfer to the Ag nanoparticles to recombine with the plasmon-induced holes produced by plasmonic absorption of Ag nanoparticles, while the VB holes remain on g-C_3_N_4_ to oxidize organic substances. Therefore, for the AgCl/Ag/g-C_3_N_4_ composites, the reduction active site is on the CB of AgCl while the oxidation active site is on the VB of g-C_3_N_4_, it is because Ag nanoparticles act as the charge separation center to form the visible light-driven AgCl/Ag/g-C_3_N_4_ Z-scheme system.

**Fig. 16:**
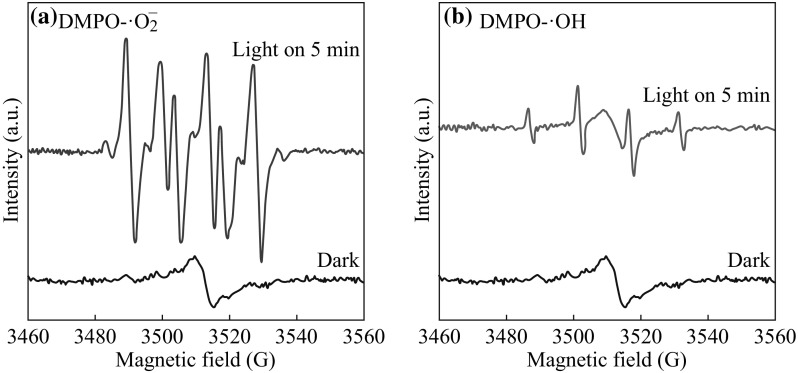
DMPO spin-trapping ESR spectra of 5 at% AgCl/Ag/g-C_3_N_4_ composite in **a** methanol dispersion (for DMPO − ·O_2_^−^), **b** aqueous dispersion (for DMPO − ·OH)

The photocatalytic mechanism is further investigated by the ESR technique. DMPO was generally applied to trapping radicals of ·O_2_^−^ and ·OH. As shown in Fig. 16, ·O_2_^−^ and ·OH radicals could be detected by the ESR under visible light irradiation. The signal of ·O_2_^−^ is much stronger than that of ·OH. Considering the band structure of g-C_3_N_4_, the VB holes (1.40 eV) from g-C_3_N_4_ cannot directly oxidize OH^−^/H_2_O into ·OH radicals (1.99 and 2.38 eV for OH^−^/·OH and H_2_O/·OH potential). The ·OH radicals should be generated via the ·O_2_^−^→H_2_O_2_ → ·OH route [43]. This fact demonstrates that ·O_2_^−^ radicals and h + are the main active species which play important roles during the photodegradation process. Based on the result, it demonstrates again that the transport process of the photoexcited carriers of the AgCl/Ag/g-C_3_N_4_ composites is in accordance with Fig. 15. Thus, the AgCl/Ag/g-C_3_N_4_ composites exhibit excellent photocatalytic performance through Z-Scheme photocatalytic mechanism under visible light.

## Conclusion

The hybrid AgCl/Ag/g-C_3_N_4_ photocatalysts were successfully fabricated by a modified deposition–precipitation method, which was effective for the control of photocatalyst morphology and size. The Ag/AgCl particles with the size of approximately 5–15 nm were evenly dispersed on the surface of g-C_3_N_4_. The AgCl/Ag/g-C_3_N_4_ composites exhibited the higher photocatalytic performance than Ag/AgCl and g-C_3_N_4_ over the degradation of Rh B or MO dyes, which was attributed to Ag nanoparticles act as the charge separation center to form the visible light-driven AgCl/Ag/g-C_3_N_4_ Z-scheme system. This study provides new insight into the design of highly efficient and stable g-C_3_N_4_-based plasmonic Z-Scheme photocatalysts and facilitates their practical application.
